# How to detect a positive response to a fluid bolus when cardiac output is not measured?

**DOI:** 10.1186/s13613-019-0612-x

**Published:** 2019-12-16

**Authors:** Zakaria Ait-Hamou, Jean-Louis Teboul, Nadia Anguel, Xavier Monnet

**Affiliations:** 1 Faculté de Médecine, Université Paris-Saclay, Le Kremlin-Bicêtre, France; 2 AP-HP, Service de médecine intensive-réanimation, Hôpital de Bicêtre, 78, rue du Général Leclerc, 94 270 Le Kremlin-Bicêtre, France; 3 Inserm UMR_S 999, Univ Paris-Saclay, Faculté de médecine, Le Kremlin-Bicêtre, France

**Keywords:** Fluid challenge, Heart rate, Pulse pressure, Pulse pressure variation, Shock index, Volume expansion

## Abstract

**Background:**

Volume expansion is aimed at increasing cardiac output (CO), but this variable is not always directly measured. We assessed the ability of changes in arterial pressure, pulse pressure variation (PPV) and heart rate (HR) or of a combination of them to detect a positive response of cardiac output (CO) to fluid administration.

**Methods:**

We retrospectively included 491 patients with circulatory failure. Before and after a 500-mL normal saline infusion, we measured CO (PiCCO device), HR, systolic (SAP), diastolic (DAP), mean (MAP) and pulse (PP) arterial pressure, PPV, shock index (HR/SAP) and the PP/HR ratio.

**Results:**

The fluid-induced changes in HR were not correlated with the fluid-induced changes in CO. The area under the receiver operating characteristic curve (AUROC) for changes in HR as detectors of a positive fluid response (CO increase ≥ 15%) was not different from 0.5. The fluid-induced changes in SAP, MAP, PP, PPV, shock index (HR/SAP) and the PP/HR ratio were correlated with the fluid-induced changes in CO, but with *r* < 0.4. The best detection was provided by increases in PP, but it was rough (AUROC = 0.719 ± 0.023, best threshold: increase ≥ 10%, sensitivity = 72 [66–77]%, specificity = 64 [57–70]%). Neither the decrease in shock index nor the changes in other indices combining changes in HR, shock index, PPV and PP provided a better detection of a positive fluid response than changes in PP.

**Conclusion:**

A positive response to fluid was roughly detected by changes in PP and not detected by changes in HR. Changes in combined indices including the shock index and the PP/HR ratio did not provide a better diagnostic accuracy.

## Background

Volume expansion is primarily aimed at increasing cardiac output (CO), with the expectation that oxygen delivery and tissue perfusion will improve consequently [1]. Nevertheless, this response of CO is inconsistent due to the curvilinearity of the Frank–Starling relationship [2].

CO is not directly monitored in every patient [3]. Its response to volume expansion is often evaluated through changes in variables used as surrogates [3]. We already demonstrated that the fluid-induced changes in CO could be very roughly monitored by arterial pulse pressure (PP) [4]. Other studies showed that the changes in PP, systolic (SAP) or mean (MAP) arterial pressure were unable to detect changes in CO [5–7]. In theory, the response of CO to fluid should be reflected by the decrease in pulse pressure variation (PPV). However, the coefficient of correlation between fluid-induced changes in CO and in PPV was only 0.6 in one study [5] and 0.27 in another one [7]. Many clinicians rely on a decrease in heart rate to assess the effects of a fluid challenge [3, 8]. However, this decrease seems to be small when observing previous studies [4].

Although changes in each of these variables may not accurately reflect the changes in CO, their combination may do so. The shock index (heart rate divided by SAP), or the ratio of PP over heart rate, which has demonstrated a strong relationship with stroke volume [9], have been proposed as non-invasive indices to assess the haemodynamic status [10]. Their possible ability to detect the effects of fluid has not been investigated yet.

Is it reasonable to use the changes in indices combining arterial pressure, heart rate or PPV in order to assess the effects of a fluid bolus on CO when the latter is not measured? This was the question we would like to answer in the present study. Taking the advantage of a large database of volume expansions performed in critically ill patients, we investigated whether combining the changes in heart rate, arterial pressure, PPV, shock index and the PP/heart rate ratio is useful for this purpose.

## Patients and methods

### Patients

This study was conducted in the medical intensive care unit of a university hospital. All patients (or next of kin) were informed about the study and agreed to participate. We retrospectively examined the data of 491 non-consecutive patients with acute circulatory failure, who had been included in previous studies [11–19]. Acute circulatory failure was defined by at least one of the following signs: (i) systolic arterial pressure ≤ 90 mmHg (or fall of systolic arterial pressure ≥ 50 mmHg in hypertensive patients) or need for vasopressor, ii) urinary flow ≤ 0.5 mL/kg/h for ≥ 2 h, (iii) heart rate ≥ 100 beats/min, (iv) skin mottling or (v) blood lactate ≥ 2 mmol/L.

### Measurements

All patients were monitored by a transpulmonary thermodilution device (PiCCO-Plus or PiCCO2 device, Pulsion Medical Systems, Feldkirchen, Germany). All patients had an internal jugular vein catheter and a thermistor-tipped arterial catheter inserted through the femoral artery.

CO was measured by transpulmonary thermodilution [17]. The average of three measurements was performed [20]. Heart rate, arterial pressure and PPV were measured from the bedside monitor (Intellivue MP70, Phillips Healthcare, Amsterdam, The Netherlands) and averaged over 15 s. Arterial elastance was calculated with the formula: elastance = 0.9 × SAP/SV, where SAP is the systolic arterial pressure and SV the stroke volume.

### Study design

Before fluid infusion, transpulmonary thermodilution was performed. Heart rate, CO (obtained from thermodilution), SAP, diastolic arterial pressure (DAP), MAP, PP and PPV (PiCCO device) were recorded.

Then, a 500-mL bolus of normal saline was infused over 10 to 30 min [21]. Immediately after end of fluid infusion, SAP, DAP, MAP, PP and PPV were recorded and a second transpulmonary thermodilution measurement was performed for measuring CO.

### Statistical analysis

A positive response to fluids was defined by an increase in CO ≥ 15% at the end of the 500-mL infusion. Normality of the data was tested by the Kolmogorov–Smirnov test. Results are expressed as mean ± SD, median [interquartile range] or mean (95% confidence interval). Comparisons between before vs. after fluid administration were assessed through a paired Student’s *t* test or a Wilcoxon test. Comparisons between fluid responders vs. fluid non-responders were assessed through a two-sample Student’s *t* test or a Mann–Whitney *U* test. Correlations were assessed by the Pearson coefficient and correlation coefficients were compared using the Fisher transformation [22].

Receiver operating characteristic (ROC) curves (with 95% confidence intervals) were constructed for testing the ability of the relative changes in heart rate, SAP, MAP, DAP, PP, PPV, the shock index and the PP/heart rate ratio to detect a positive response to fluid. For PPV, absolutes values were taken into account, while for heart rate, SAP, MAP, DAP, PP, the shock index and the PP/heart rate ratio, both absolute values and percent changes were considered. The best diagnostic thresholds were defined as those providing the highest Youden index. The areas under the ROC curves (AUROC) were compared using a Hanley–McNeil test.

For testing the diagnostic ability of combined indices, we performed two different analyses. First, we built a combined index, which was considered as positive if the value taken by changes in heart rate, in PP and in PPV were all above their respective best diagnostic threshold found by the previous ROC curve analysis, and negative if one or more of the variables was below its best diagnostic threshold. This combined index was submitted to a ROC curve analysis. Second, we performed a stepwise logistic regression, with a positive response to fluid as the dependent variable and the percent changes in heart rate, PP and PPV as independent variables.

The primary analysis was performed after excluding the far outliers of the database. A far-out value was defined as a value that was smaller than the lower quartile minus 3 times the interquartile range, or larger than the upper quartile plus 3 times the interquartile range [23]. The analysis was also performed on the whole population. A *p* value ≤ 0.05 was considered statistically significant. The statistical analysis was performed by using MedCalc8.1.0.0 (Mariakerke, Belgium).

## Results

### Patients

The characteristics at baseline of the 491 included patients are summarised in Table 1. PPV had been recorded in 358 patients. Thirty-seven (10%) patients in whom PPV had been measured presented neither atrial fibrillation, nor spontaneous breathing nor acute respiratory distress syndrome (ARDS), i.e. conditions in which PPV interpretation is not valid [2]. In particular, the proportion of patient ventilated with a tidal volume ≤ 8 mL/kg of predicted body weight was 94%. No patient presented right ventricular failure. Fluid was administered over 10 min in 204 patients and over 30 min in 287 patients.

**Table 1:** Patient characteristics at baseline

Gender (no. of patients, F/M)	204/287
Age (mean ± SD, years)	63 ± 13
Cardiac rhythm (no. of patients, %)	
Sinus	397 (81)
Atrial fibrillation	88 (18)
Atrial extrasystoles	6 (1)
SAPSII (mean ± SD)	63 ± 13
Type of shock (no. of patients, %)	
Septic	347 (71)
Hypovolemic	100 (20)
Cardiogenic	23 (5)
Vasoplegic (non-septic)	21 (4)
Reasons for fluid administration (no of patients, %)^a^	
Hypotension	101 (21)
Tachycardia	201(41)
Oliguria	220 (45)
Skin mottling	54 (11)
Tissue hypoxia	264 (54)
Mechanical ventilation (no. of patients, %)	327 (67)
Tidal volume (mean ± SD, mL/kg of PBW)	6.3 (0.8)
ARDS (no. of patients, %)	295 (60)
Sedation (no. of patients, %)	319 (65)
Lactate (mean ± SD, mmol/L)	3.1 (1.2)
Patients receiving NE at baseline (no. of patients, %)	346 (71)
NE dose at baseline (median [25–75% IQ] μg/kg/min	0.7 [0.6–1.6]

*N* = 491

*ARDS* acute respiratory distress syndrome, *NE* norepinephrine, *SAPS II* simplified acute physiology score, *IQ* interquartile

^a^Several reasons might have been present in a patient simultaneously

### Haemodynamic effects of volume expansion

In the whole population, volume expansion increased CO by 22 ± 23%. It increased by more than 15% in 275 (56%) “fluid-responders”. In fluid responders, CO increased by 36 ± 21% and heart rate decreased by 2 ± 9%. In these patients, SAP, MAP, PP and DAP increased by 19 ± 22%, 16 ± 21%, 27 ± 35% and 14 ± 38%, respectively (Table 2).

**Table 2:** Changes in haemodynamic variables induced by volume expansion

	Before volume expansion	After volume expansion
Heart rate (mean ± SD, beats/min)		
Non-responders	92 ± 21	91 ± 21*
Responders	99 ± 22^#^	97 ± 20*^#^
Systolic arterial pressure (mean ± SD, mmHg)		
Non-responders	110 ± 22	117 ± 25*
Responders	109 ± 22	128 ± 25*^#^
Diastolic arterial pressure (mean ± SD, mmHg)		
Non-responders	55 ± 12	57 ± 13*
Responders	53 ± 11	59 ± 13*
Mean arterial pressure (mean ± SD, mmHg)		
Non-responders	71 ± 16	75 ± 18*
Responders	69 ± 14	79 ± 17*
Arterial pulse pressure (mean ± SD, mmHg)		
Non-responders	55 ± 17	60 ± 19*
Responders	56 ± 18	69 ± 21*^#^
Shock index (mean ± SD, beats/min/mmHg)		
Non-responders	0.87 ± 0.28	0.81 ± 0.25*
Responders	0.95 ± 0.36^#^	0.79 ± 0.22*
Cardiac index (mean ± SD, L/min/m^2^)		
Non-responders	3.4 ± 1.2	3.6 ± 1.3*
Responders	2.8 ± 1.0^#^	3.7 ± 1.2*
Arterial elastance (mean ± SD, mmHg/mL)		
Non-responders	1.71 ± 0.74	1.64 ± 0.73*
Responders	2.27 ± 1.08^#^	1.61 ± 0.61*

*N* = 275 in responders and 216 in non-responders

* *p* < 0.05 vs. Before volume expansion (comparisons in rows); ^#^ *p* < 0.05 vs. Non-responders (comparisons in columns)

### Ability of changes in arterial pressure to detect the fluid-induced changes in CO

**Fig. 1:**
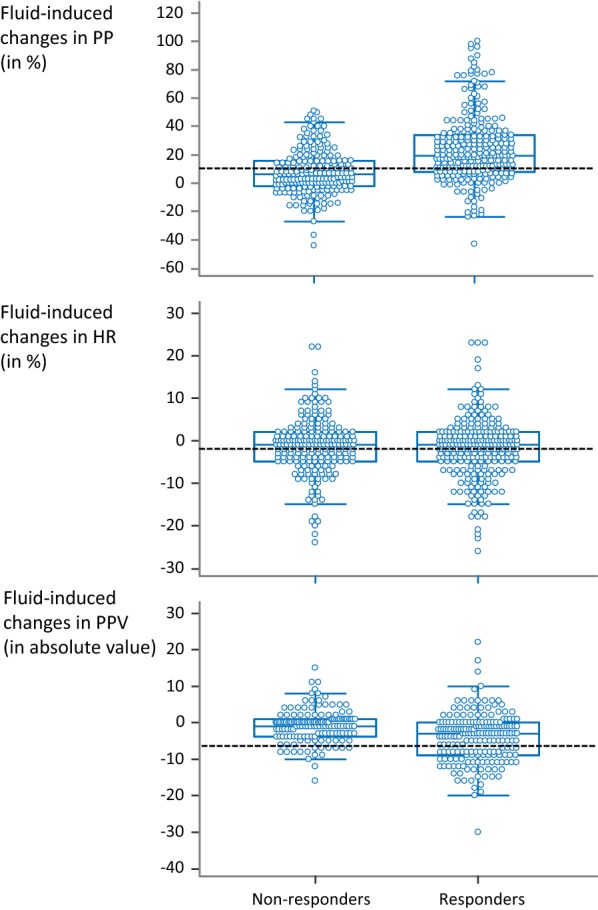
Individual values of the fluid-induced changes in arterial pulse pressure (PP), heart rate (HR) and pulse pressure variation (PPV) in patients in whom volume expansion increased cardiac output ≥ 15% (responders) and in the other ones. Dashed lines represent the best threshold for detecting a positive fluid response found by statistical analysis. Outliers were excluded from analysis

**Fig. 2:**
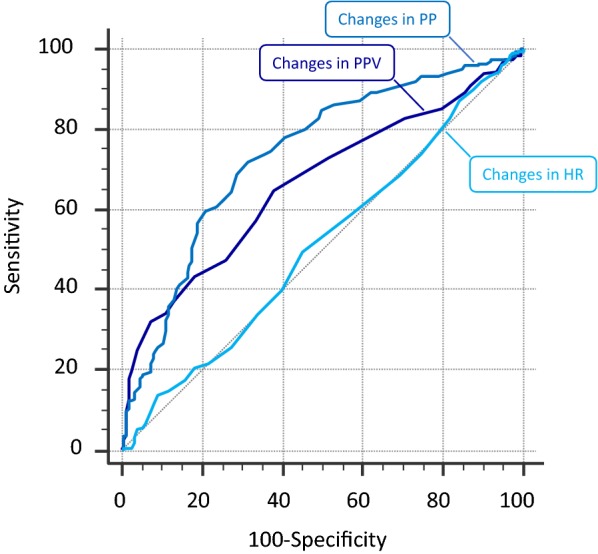
Receiver operating characteristic curves for changes in arterial pulse pressure (PP), heart rate (HR) and pulse pressure variation (PPV) to detect a fluid-induced increase in cardiac index ≥ 15%. Outliers were excluded from analysis

The coefficient of correlation between the fluid-induced changes in arterial pressure values and changes in CO after exclusion of outliers is provided in Table 3. The ability of these changed to detect a positive fluid response are described in Table 3 and Figs. 1 and 2. The best AUROC was observed for the changes in PP, with a best diagnostic threshold of 10%. The ability of changes in PP to detect a positive fluid response varying the threshold is described in Additional file 1: Table S1 for percent changes and in Additional file 1: Table S2 for changes in absolute values. The results obtained without excluding outliers are provided in Additional file 1: Table S3. The diagnostic ability was similar in patients in whom fluid administration was decided because of hypotension (SAP ≤ 90 mmHg) (*n* = 101) (AUROC: 0.575 ± 0.058, *p* = 0.19 vs. 0.500) compared to the other ones. The AUROC for changes in PP to detect a positive fluid response was similar in patients in whom fluid was infused over 10 min and those in whom it was infused over 30 min (0.680 ± 0.041 vs. 0.726 ± 0.030, *p* = 0.36). It was also similar in patients older and younger than 60 years old (0.708 ± 0.031 vs. 0.745 ± 0.037, *p* = 0.44).

**Table 3:** Diagnostic ability of the changes in haemodynamic variables to diagnose a fluid-induced increase in cardiac output ≥ 15%

	*r* vs. changes in CO	*p* value for the correlation	Sensitivity	Specificity	Positive PV	Negative PV	Positive LR	Negative LR	Best threshold	AUROC	*p* value for AUROC vs. 0.50	*n*
Decrease in HR (in %)	–	0.06	49 (43–55)	57 (50–64)	59 (54–63)	47 (43–54)	1.1 (0.9–1.4)	0.9 (0.8–1.1)	< − 2%	0.529 ± 0.026*	0.27	479
Increase in SAP (in %)	0.38	< 0.0001	65 (58–70)	66 (66–73)	71 (66–75)	60 (55–64)	1.9 (1.6–2.4)	0.5 (0.4–0.6)	> 9%	0.697 ± 0.024	< 0.001	482
Increase in MAP (in %)	0.33	< 0.0001	58 (52–64)	70 (63–76)	71 (66–75)	57 (53–61)	1.9 (1.5–2.4)	0.6 (0.5–0.7)	> 8%	0.654 ± 0.026*	< 0.001	484
Increase in DAP (in %)	–	0.84	53 (46–60)	52 (45–59)	54 (49–58)	51 (46–56)	1.1 (0.9–1.3)	0.9 (0.7–1.1)	> 31%	0.509 ± 0.029*	0.77	410
Decrease in shock index (in %)	− 0.35	< 0.0001	66 (60–72)	64 (57–70)	70 (66–74)	60 (55–64)	2.0 (1.6–2.4)	0.5 (0.4–0.6)	< − 9%	0.688 ± 0.024	< 0.001	479
Increase in PP/HR ratio (in %)	0.33	< 0.0001	52 (46–58)	79 (72–83)	76 (70–80)	56 (52–59)	2.4 (1.8–3.2)	0.6 (0.5–0.7)	> 41%	0.668 ± 0.024	< 0.001	479
Increase in PP (in %)	0.38	< 0.0001	72 (66–77)	64 (57–70)	71 (67–75)	65 (60–70)	1.8 (1.5–2.2)	0.4 (0.4–0.5)	> 10%	0.719 ± 0.023	< 0.001	478
Decrease in PPV (in abs val) (all)	− 0.24*	< 0.0001	64 (57–71)	63 (54–70)	70 (64–74)	57 (52–63)	1.7 (1.4–2.2)	6.0 (0.5–0.7)	< − 2 points	0.663 ± 0.028*	< 0.001	357
Decrease in PPV (in abs val) (without arrhythmia, spontaneous breathing, and ARDS)	− 0.24*	< 0.0001	48 (28–69)	100 (73–100)	100 (75–100)	48 (39–57)	–	1.0 (1.0–1.0)	< − 7 points	0.750 ± 0.080	< 0.001	37
Decrease in PPV (in abs val) (without arrhythmia and spontaneous breathing)	0.56	< 0.0001	33 (23–44)	86 (77–93)	74 (59–84)	54 (50–58)	2.5 (1.3–4.9)	0.8 (0.6–0.9)	< − 8 points	0.532 ± 0.045	0.48	160

*AUROC* area under the receiver operating characteristic curve, *CO* cardiac output, *DAP* diastolic arterial pressure, *HR* heart rate, *LR* likelihood ratio, *MAP* mean arterial pressure, *n* number of patients included in the analysis after exclusion of outliers, *PP* pulse pressure, *PPV* pulse pressure variation, *PV* predictive value, *SAP* systolic arterial pressure, *Vt* tidal volume

* *p* < 0.05 vs. results obtained for changes in PP (in %). Values are expressed as mean ± standard deviation or mean (95% confidence interval)

### Ability of the changes in PPV to detect the fluid-induced changes in CO

Considering all the 358 patients in whom PPV had been measured, after exclusion of outliers, the correlation between fluid-induced changes in PPV (in absolute value) and changes in CO (in %) was significant but lower than for the fluid-induced changes in PP (Table 3).

The fluid-induced changes in PPV (in absolute value) were able to detect a positive fluid response (Table 3, Figs. 1 and 2). The diagnostic ability depending on the threshold chosen for changes in PPV in absolute values is reported in Additional file 1: Table S4. The diagnostic accuracy was not better than that of changes in PP. The best diagnostic threshold was a decrease in PPV ≥ 2 points (Table 3, Figs. 1 and 2). The results obtained in patients without atrial fibrillation, spontaneous breathing activity and ARDS (*n* = 37) are shown in Table 3 and the results without excluding outliers in Additional file 1: Table S3. The diagnostic ability depending on the threshold chosen for changes in PPV in absolute values in patients without atrial fibrillation, spontaneous breathing activity and ARDS (*n* = 37) is reported in Additional file 1: Table S5. The AUROC for changes in PPV to detect a positive fluid response was similar in patients in whom fluid was infused over 10 min and over 30 min (0.562 ± 0.059 vs. 0.502 ± 0.040, *p* = 0.40).

### Ability of changes in heart rate to detect the fluid-induced changes in CO

The correlation between fluid-induced changes in heart rate (in %) and changes in CO (in %) after exclusion of outliers was not significant (Table 3). The fluid-induced changes in heart rate (in %) were not able to detect a positive response to fluid (Table 3, Figs. 1 and 2). The ability of changes in heart rate to detect a positive response to fluid depending on the threshold is described in Additional file 1: Table S6 for percent changes and in Additional file 1: Table S7 for changes in absolute values. The results obtained without excluding outliers are provided in Additional file 1: Table S3.

The diagnostic ability of the fluid-induced decrease in heart rate was not different when taking into account only the patients with hypovolemic shock (*n* = 99), the patients with sinus rhythm (*n* = 390) or the patients without sedation (*n* = 169) (AUROC: 0.509 ± 0.06, 0.532 ± 0.03 and 0.541 ± 0.05, respectively, all not different from 0.500). It was also similar in patients older and younger than 60 years old (AUROC: 0.599 ± 0.03 and 0.587 ± 0.04, respectively, *p* = 0.81), and after excluding the 35 patients with previous administration of beta-blockers (AUROC: 0.525 ± 0.03). The diagnostic ability was also similar in patients with tachycardia before fluid administration (*n* = 197) (AUROC: 0.547 ± 0.41). The AUROC for changes in heart rate to detect a positive fluid response was similar in patients in whom fluid was infused over 10 min and those in whom it was infused over 30 min (0.524 ± 0.043 vs. 0.563 ± 0.034, *p* = 0.48).

### Ability of changes in shock index to detect the fluid-induced changes in CO

The correlation between the fluid-induced changes in shock index (in %) and the fluid-induced changes in CO (in %) after exclusion of outliers was significant, but the correlation coefficient was significantly lower than for the fluid-induced changes in PP (Table 3).

Considering the whole population, the fluid-induced changes in shock index (in %) were able to detect a positive fluid response (Table 3). The best diagnostic threshold was a decrease in shock index by more than 9% (Table 3). The ability of changes in shock index depending on the threshold are described in Additional file 1: Table S8 for percent changes and in Additional file 1: Table S9 for changes in absolute values. The results obtained without excluding outliers are provided in Additional file 1: Table S3. The AUROC for changes in shock index to detect a positive fluid response was similar in patients in whom fluid was infused over 10 min and those in whom it was infused over 30 min (0.598 ± 0.041 vs. 0.529 ± 0.029, *p* = 0.17).

### Ability of changes in the PP/heart rate ratio to detect the fluid-induced changes in CO

The correlation between the fluid-induced changes in the PP/heart rate ratio (in %) and the fluid-induced changes in CO (in %) after exclusion of outliers was significant, but the correlation coefficient was the same as for the fluid-induced changes in PP (Table 3).

Considering the whole population, the fluid-induced changes in the PP/heart rate ratio (in  %) were able to detect a positive fluid response (Table 3). The best diagnostic threshold was an increase in the PP/heart rate ratio by more than 41% (Table 3). The ability of changes in the PP/heart rate ratio varying the threshold are described in Additional file 1: Table S10 for percent changes and in Additional file 1: Table S11 for changes in absolute values. The AUROC for changes in the PP/heart rate ratio to detect a positive fluid response was similar in patients in whom fluid was infused over 10 min and those in whom it was infused over 30 min (0.625 ± 0.41 vs. 0.667 ± 0.032, *p* = 0.37).

### Ability of index combining changes in heart rate, PP, shock index and PPV to detect the fluid-induced changes in CO

Using logistic regression, only the changes in PP were independently associated with a positive response to fluid infusion (odds ratio: 1.038 [1.025–1.052]). The analysis performed by considering each variable as either positive or negative by taking the threshold found at univariate analysis, the only combination that provided a significant AUROC was a decrease in heart rate > 2% coupled with an increase in PP ≥ 10%. It was inferior to the one provided by the changes in PP (Additional file 1: Table S12).

## Discussion

We showed that the effects of volume expansion on CO cannot be detected by the simultaneous changes in heart rate. The changes in PP only roughly detected a positive fluid response. The changes in PPV reliably detected the fluid response in patients fulfilling the conditions of PPV interpretation. The changes in shock index or in any other indices combining the changes in these variables did not provide a better diagnostic accuracy. These retrospective results suggest that one needs to directly measure CO to detect a positive fluid response.

Because they are invasive, costly or because they require skills and time, CO-monitoring devices are not always used by clinicians [3], though it is recommended to monitor CO in patients with acute circulatory failure that resists to treatment [20]. This might also be the case in high-risk surgical patients [24], in spite of the benefit demonstrated by CO monitoring in these patients [25]. In such cases, clinicians only rely on surrogates to assess the fluid effects on CO [8]. Determining the reliability of such surrogates is important for the clinical practice.

The fact that the changes in arterial pressure, including PP, do not reliably reflect the simultaneous changes in CO has been already demonstrated by several studies [4, 6]. However, a large number of clinicians do not monitor CO directly during fluid administration [8] and still use arterial pressure and other simple haemodynamic indices to estimate the fluid-induced CO changes. Not less than 24–26% of respondents relied on changes in heart rate to evaluate the response to a fluid challenge [3, 8]. Some other combined variables are used. The value of the shock index has been emphasised in a recent recommendation regarding monitoring during fluid infusion in resource-limited settings [26]. The PP/heart rate ratio has been found to be related to stroke volume [9] and to predict massive transfusions in severe trauma [27]. The changes in PPV have been poorly investigated. In our previous study [4], the number of patients did not allow us to investigate the diagnostic accuracy of changes in PPV in the subgroup of patients in whom its interpretation was valid. Finally, the number of patients included in previous studies did not allow their authors to exclude outliers without affecting the power of analysis.

Increasing CO should physiologically decrease the sympathetic stimulation and should be accompanied by a decrease in heart rate. We observed such a decrease but it was of small magnitude, it was poorly correlated with changes in CO, and its amplitude was greatly variable from a patient to another. Eventually, it was impossible to define a threshold that accurately detected a positive response to fluid. A good specificity was achieved only for extreme changes. The ability of heart rate changes to detect the fluid response of CO was still disappointing when excluding patients receiving sedatives or with atrial fibrillation, when including only patients with hypovolemic shock or patients with tachycardia before fluid infusion. We conclude that relying on changes in heart rate for assessing the effects of a fluid bolus on CO is not reasonable.

The variable that was the best for detecting changes in CO was PP, but its diagnostic accuracy was only rough. In fact, the relationship between CO and peripheral PP changes is not straightforward since it depends on heart rate (relationship between CO and stroke volume), on the arterial compliance (relationship between stroke volume and PP) and on the pulse wave amplification from the aorta to the periphery. The changes in heart rate were small in our study on average, suggesting that this phenomenon plays a minor role. The arterial blood pressure measurements were performed at the level of the femoral artery, which minimises the importance of the pulse wave amplification phenomenon. It is thus likely that the arterial compliance must explain the discrepancy we observed. As a matter of fact, fluid infusion may change the arterial compliance and this phenomenon might be independent from the arterial properties at baseline [28]. For a similar fluid-induced increase in stroke volume, PP may have increased to a larger extent in patients in whom the arterial compliance decreased than in patients in whom it remained normal. Our study quantifies the amplitude of the error that is made when the effects of a fluid bolus are monitored only with PP. This resulted almost in one-third of false positives and false negatives, which is not acceptable in severe patients, in whom the effects of treatments should be accurately assessed.

These results corroborate previous observations during fluid infusion [4, 5] or passive leg raising [29]. Pierrakos et al. [6] showed even no correlation between fluid-induced changes in PP and in CO. This discrepancy might be explained by the fact that they measured PP in the radial artery while we did it at the iliac level [6], with a different pulse wave amplification.

The changes in MAP tended to have a lower accuracy than those in PP to detect the fluid effects. The changes in MAP are dissociated from the changes in CO due to the sympathetic modulation, which tends to maintain MAP constant while CO varies [30]. Surprisingly, some other authors did not report different diagnostic ability between changes in PP and in MAP [5], a result that goes against the expected physiology.

Of note, we observed that the rate of cases in which PPV was valid was 10%. It is higher than the 1% [31] or 2% [32] prevalence rates reported by some authors. Nevertheless, these authors calculated this prevalence in all patients hospitalised in an intensive care unit at 1 day [31, 32]. This was meaningless, because the question of PPV validity is pertinent only in patients in whom one needs to test fluid responsiveness. In particular, including into this analysis patients without circulatory failure dramatically increases the proportion of patients with spontaneous breathing, while the question of administering fluid would never be asked in such patients. Our results are much more informative, like those reported during the first 24 h of hospitalisation of critically ill patients [33], or in patients with an unstable haemodynamic event [34].

Finally, taking advantage of the large number of cases, we could explore indices combining the changes in several variables. Neither the shock index nor the PP/heart rate ratio was better than PP for detecting the fluid effects on CO. It was either not better for other combinations of changes in haemodynamic variables and, using logistic regression, no model could be found for detecting a positive response to fluid from heart rate, PP and PPV. Some of these combined indices did even not provide any significant relationship with changes in CO.

The implications of the present study for the practice are important. It shows that monitoring the effects of a fluid challenge on CO through blood pressure, as reported in 67% of cases in the Fenice survey [3], is not accurate. Using the fluid-induced changes in heart rate (24% of instances in Fenice [3]) is even worse. Our study is an evidence-based support for the recommendation to measure stroke volume in patients with acute circulatory failure which persists despite adequate fluid resuscitation [35]. In such severe patients, mistakes in fluid therapy may have dramatic consequences. Overestimating these effects on CO may lead to under-resuscitation. Even worse would be the consequences of underestimating these effects, leading to continue fluid infusion without any benefit. The harm created by fluid overload in critically ill patients is now well established [36], especially in case of ARDS or sepsis [37].

This study has limitations. First, it was retrospective. Nevertheless, the population was made of patients included into prospective trials. Second, using data from different studies inevitably introduces the problem of heterogeneity. In particular, the time over which fluid administration was performed was different between patients, while the efficacy of a fluid bolus is only transient [38]. Nevertheless, the AUROC for the changes in the investigated variables were similar among patients with infusions over 10 min and over 30 min. Third, the central venous pressure was not measured in a large majority of the included studies, such that it was not integrated to analysis. Its changes, in combination with other variables, might be interesting for tracking CO changes, but this should be studied. Fourth, as we included a general population of critically ill patients, one cannot exclude that the detection of haemodynamic trends could be different if focusing on specific groups and defined indications of the fluid bolus. Finally, we could not directly assess whether monitoring CO rather than surrogates reduces the amount of fluid over or under-administered. This remains to be tested.

## Conclusion

The effects of a fluid challenge on CO cannot at all be detected by the changes in heart rate. The changes in PP and in PPV only provide a rough estimation. A reliable assessment of the effects of volume expansion on CO requires a direct measurement of it. These data argue for monitoring CO in patients who require a precise haemodynamic assessment.

## Supplementary information

**Additional file 1.** Tables S1–S12 and Figure S1.

## Data Availability

I agree to the terms of the BioMed Central License Agreement and Open data policy.

## Abbreviations

ARDS: acute respiratory distress syndrome
AUROC: area under the receiver operating characteristic curve
CO: cardiac output
DAP: diastolic arterial pressure
MAP: mean arterial pressure
PP: pulse pressure
PPV: pulse pressure variation
ROC: receiver operating characteristic
SAP: systolic arterial pressure
