# Supplementary material for: How to detect a positive response to a fluid bolus when cardiac output is not measured?
Source: Ann Intensive Care. 2019 Dec 16;9:138. doi: 10.1186/s13613-019-0612-x (PMC6915177; doi:10.1186/s13613-019-0612-x)

How to detect a positive response to a fluid bolus when cardiac output is not measured?

Zakaria AIT-HAMOU, MD^1,2,3^

Jean-Louis TEBOUL MD, PhD^1,2,3^

Nadia ANGUEL, MD^1,2,3^

Xavier MONNET, MD, PhD^1,2,3^

Additional file

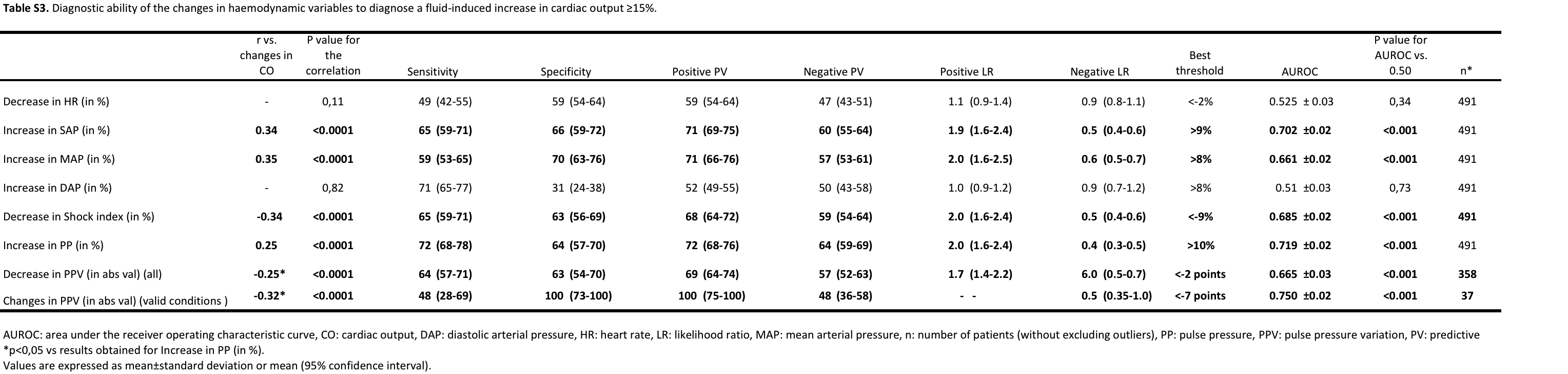

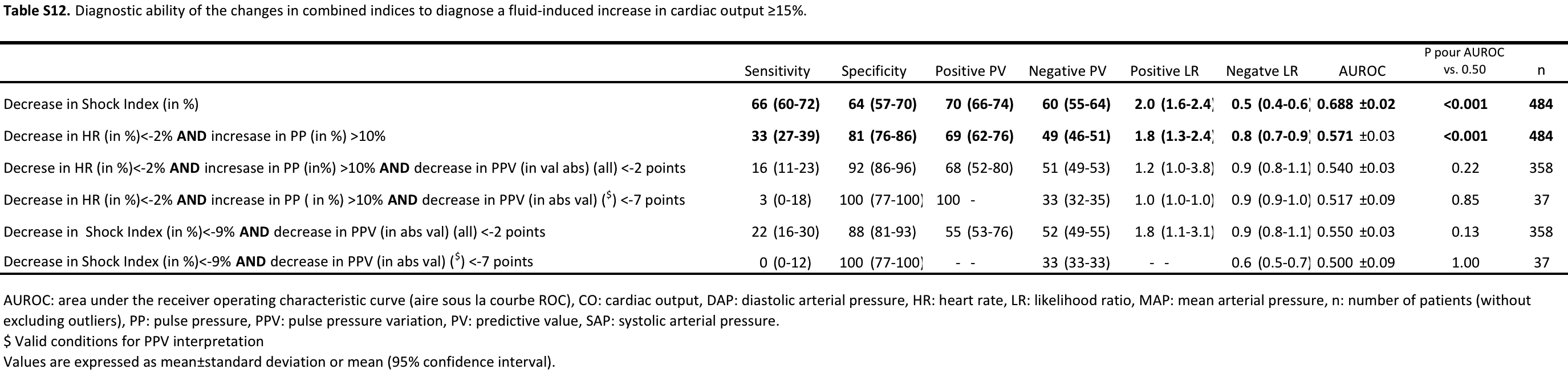


Legend of figures

Figure S1.

Receiver operating characteristic curves for changes in arterial pulse pressure (PP), heart rate (HR) and pulse pressure variation (PPV) to detect a fluid-induced increase in cardiac index ≥15% in patients without spontaneous breathing and cardiac arrhythmias.

Outliers were excluded from analysis.


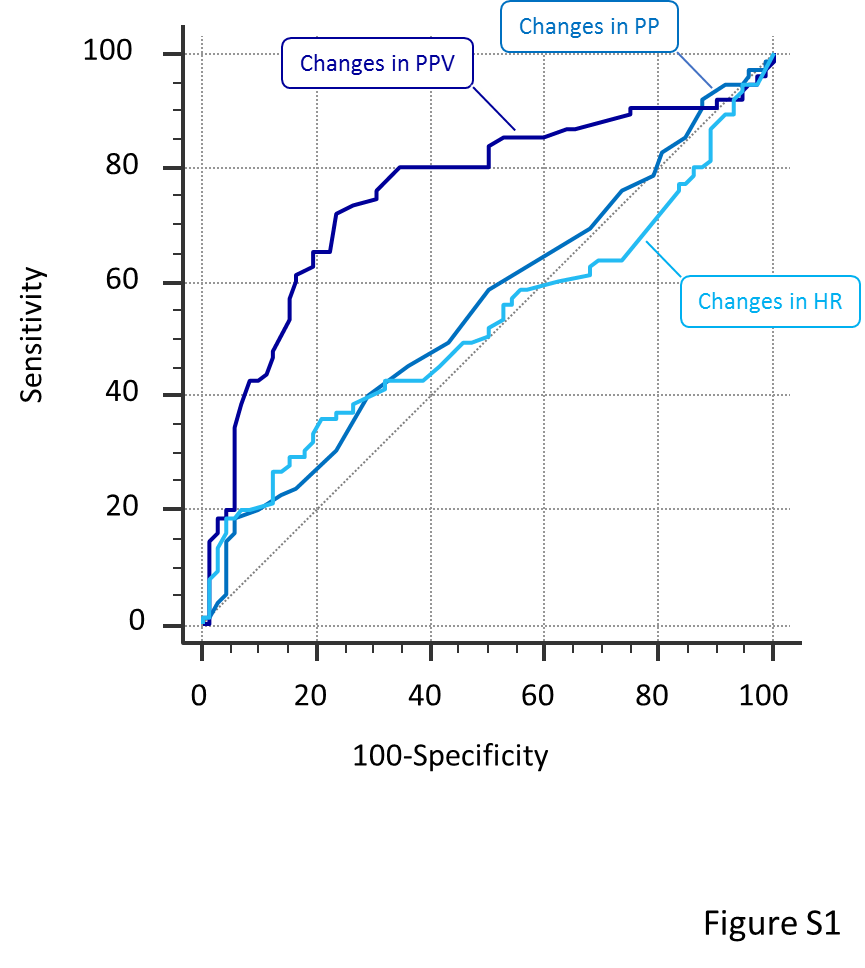

Supplement: Supplementary file 1 — Additional file 1. Tables S1–S12 and Figure S1. [file 13613_2019_612_MOESM1_ESM.docx]
